# Double-channel gastroscopy-assisted holmium laser lithotripsy for giant common bile duct stones with a fistula after Billroth Ⅱ​ reconstruction

**DOI:** 10.1055/a-2938-1466

**Published:** 2026-08-28

**Authors:** Linfu Zheng, Jin Zheng, Zhilin Liu, Dazhou Li

**Affiliations:** 1 Department of Gastroenterology 608776 Fuzong Clinical Medical College of Fujian Medical UniversityFuzhouFujianChina; 2 Department of Gastroenterology 85116 The 900th Hospital of Joint Logistic Support Force, Chinese People's Liberation ArmyFuzhouFujianChina

Endoscopic retrograde cholangiopancreatography (ERCP) is technically challenging in patients with Billroth II anatomy because reaching and selectively cannulating the papilla can be difficult, and the risk of procedure-related perforation is increased. 1​ ​2​ Management is particularly demanding when the common bile duct (CBD) contains a giant stone.

**Fig. 1:**
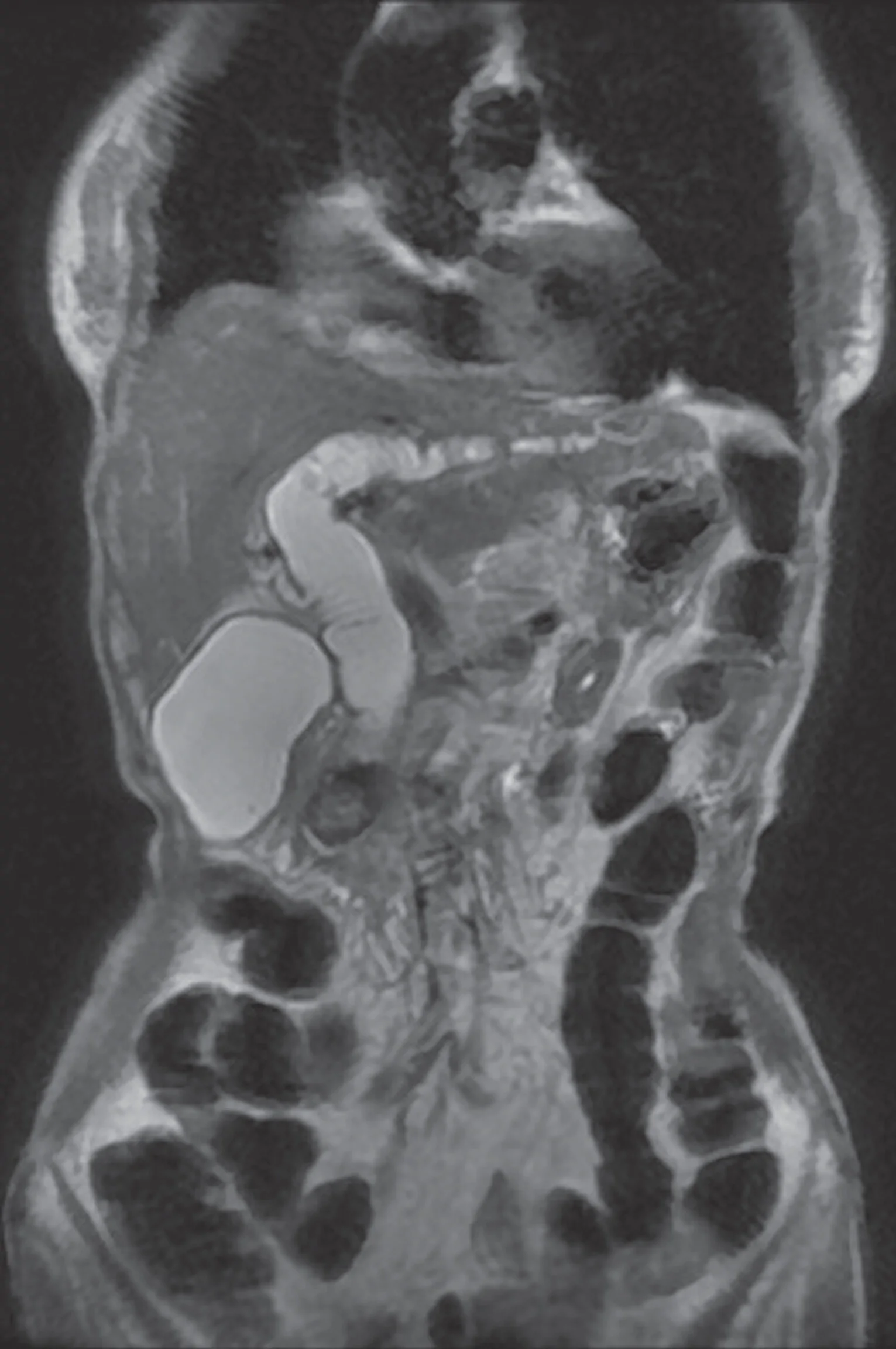
Upper abdominal magnetic resonance image showing a 2×3-cm stone in the common bile duct.

An 84-year-old woman presented with a 2-day history of right upper quadrant pain, abdominal distension, chills, and fever (a maximum temperature of 39.5°C). Computed tomography performed at another hospital demonstrated cholelithiasis and an obstructing distal CBD stone. Upon admission, laboratory tests showed a white blood cell count of 11.41×10 ^9^ /L, a neutrophil percentage of 87.0%, a neutrophil count of 9.94×10 ^9^ /L, a procalcitonin level of 6.35 ng/mL, and a C-reactive protein level of 137.4 g/L. Liver biochemical tests were also abnormal: alanine aminotransferase, 115.5 U/L; aspartate aminotransferase, 84.5 U/L; alkaline phosphatase, 653.9 U/L; γ-glutamyl transferase, 1219.3 U/L; total bilirubin, 30.6 μmol/L; direct bilirubin, 20.2 μmol/L; and total bile acids, 11.65 μmol/L. Upper abdominal magnetic resonance imaging showed a 2×3-cm CBD stone ( Fig. 1 ). Ten years earlier, the patient had undergone distal gastrectomy with Billroth II reconstruction for a bleeding gastric ulcer.

**Fig. 2:**
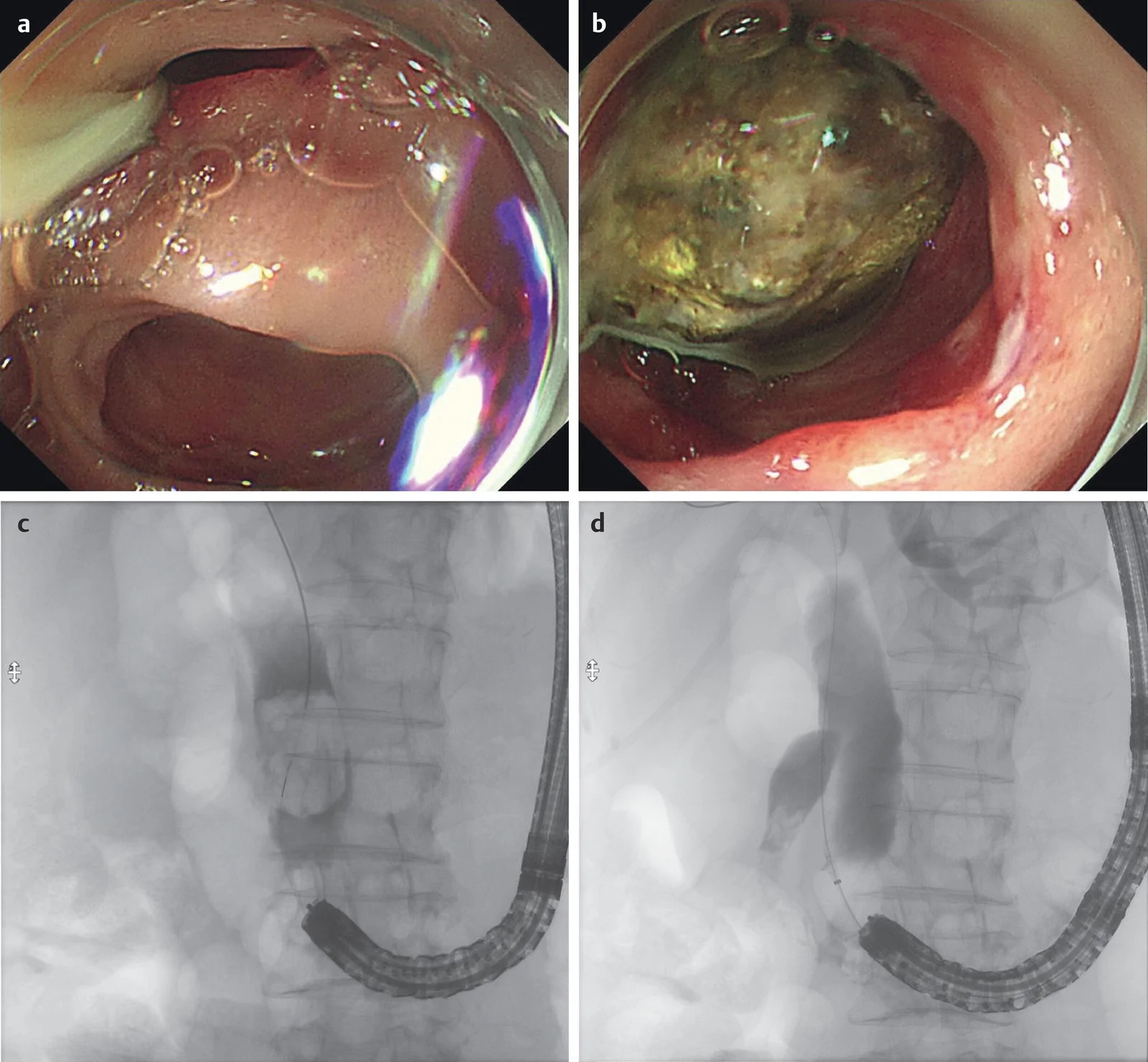
( **a** ) Purulent drainage from the fistulous opening immediately proximal to the major papilla. ( **b** ) A stone visible through the fistulous opening. ( **c** ) Endoscopic retrograde cholangiography showing a 2×3-cm filling defect in the distal common bile duct. ( **d** ) Final endoscopic retrograde cholangiography showing complete clearance of the common bile duct.

ERCP was undertaken for biliary decompression and stone removal. A forward-viewing gastroscope fitted with a transparent distal attachment was advanced into the afferent limb instead of a duodenoscope to facilitate safer intubation in the surgically altered anatomy. The major papilla was identified in the afferent limb. A fistulous opening immediately proximal to the papilla was draining pus ( Fig. 2a ), and a stone was directly visible through the opening ( Fig. 2b ). The biliary tract was cannulated using a CleverCut 3 V single-use three-lumen sphincterotome (KD-V411M-0725; Olympus Medical Systems Corp., Tokyo, Japan) over a single-use hydrophilic guide wire for ERCP (0.035 inch×450 cm; JHY-GW-88-450-C1; Changzhou Jiuhong Medical Instrument Co., Ltd, Changzhou, China). Cholangiography demonstrated marked CBD dilation and a 2×3-cm filling defect in the distal CBD ( Fig. 2c ). Conventional extraction and lithotripsy were unsuccessful because of the stone's size. Holmium:yttrium-aluminum-garnet (Ho:YAG) laser lithotripsy was therefore attempted using a holmium laser therapeutic apparatus (HZ-40; Guangzhou Potent Medical Equipment Joint-Stock Co., Ltd, Guangzhou, China) and a laser fiber (HZ-40; Guangzhou Potent Medical Holmium Laser Therapeutic Apparatus and a laser fiber Equipment Joint-Stock Co., Ltd, Guangzhou, China). However, when the fiber was advanced through the working channel, the stone repeatedly migrated proximally, preventing stable laser targeting.

The endoscope was then exchanged for a GIF-2TQ260M dual-channel multibending video gastroscope with two 3.2-mm working channels (Olympus Medical Systems Corp., Tokyo, Japan). Through one channel, a single-use stone extraction balloon (MTN-SRB-T-16/20; Micro-Tech [Nanjing] Co., Ltd, Nanjing, China) was positioned proximal to the stone and used to reposition and stabilize it in the distal CBD. Through the second channel, the Ho:YAG laser fiber was applied under direct endoscopic visualization at 3.0 J per pulse and 30 W. After fragmentation, the stone fragments were removed using a memory basket 7 Fr hard wire (3×6 cm; MWB-3×6; Cook Medical LLC, Bloomington, Indiana, USA) and the extraction balloon ( Video 1 ). No residual stones were evident on endoscopy or cholangiography ( Fig. 2d ). After the procedure, the patient received intravenous antibiotics, fluids, and nutritional support. Three days later, the white blood cell count had decreased to 5.18×10 ^9^ /L, the neutrophil count to 3.73×10 ^9^ /L, the C-reactive protein level to 20.1 mg/L, and the procalcitonin level to 0.45 ng/mL. Serum amylase and liver biochemical test results were normal. Her abdominal pain resolved, she remained afebrile, and she was discharged without adverse events.

**Video 1** Dual-channel gastroscope-assisted Ho:YAG laser lithotripsy for a giant common bile duct stone in a patient with a biliary fistula after Billroth II reconstruction.


In patients with Billroth II anatomy, a giant CBD stone, a biliary fistula, and suppurative cholangitis, and dual-channel gastroscopy may provide an effective platform for laser lithotripsy when standard methods fail. An extraction balloon introduced through one working channel can reposition and immobilize the stone, while a laser fiber introduced through the other channel enables simultaneous, stable fragmentation. This coordinated approach may improve targeting and procedural efficiency.

Endoscopy_UCTN_Code_TTT_1AR_2AL
